# The Genome of the Yellow Mealworm, *Tenebrio molitor*: It’s Bigger Than You Think

**DOI:** 10.3390/genes14122209

**Published:** 2023-12-14

**Authors:** Brenda Oppert, Aaron T. Dossey, Fu-Chyun Chu, Eva Šatović-Vukšić, Miroslav Plohl, Timothy P. L. Smith, Sergey Koren, Morgan L. Olmstead, Dewey Leierer, Gail Ragan, J. Spencer Johnston

**Affiliations:** 1 USDA Agricultural Research Service, Center for Grain and Animal Health Research, 1515 College Ave., Manhattan, KS 66502, USA; molmste@ncsu.edu (M.L.O.); gail.ragan@usda.gov (G.R.); 2 All Things Bugs LLC, 755 Research Parkway, Suite 465, Oklahoma City, OK 73130, USA or aaron.t.dossey@gmail.com (A.T.D.); cchu@fl70inc.com (F.-C.C.); 3 Invertebrate Studies Institute, 2211 Snapper Ln, Midwest City, OK 73130, USA; 4 Division of Molecular Biology, Ruđer Bošković Institute, Bijenička 54, 10000 Zagreb, Croatia; eva.satovic.vuksic@irb.hr (E.Š.-V.); plohl@irb.hr (M.P.); 5 USDA ARS U.S. Meat Animal Research Center, Clay Center, NE 68933, USA; tim.smith2@usda.gov; 6 Genome Informatics Section, Computational and Statistical Genomics Branch, National Human Genome Research Institute, National Institutes of Health, Bethesda, MD 20894, USA; 7 Department of Molecular Biology and Biophysics, Kansas State University, Manhattan, KS 66506, USA; leierer@ksu.edu; 8 Department of Entomology, Texas A&M University, College Station, TX 77843, USA; j.johnston@ag.tamu.edu

**Keywords:** CRISPR Cas9 gene editing, insects as food and feed, insect genomics and genetics, stored product insect, *Tenebrio molitor*, yellow mealworm

## Abstract

Background: Insects are a sustainable source of protein for human food and animal feed. We present a genome assembly, CRISPR gene editing, and life stage-specific transcriptomes for the yellow mealworm, *Tenebrio molitor*, one of the most intensively farmed insects worldwide. Methods: Long and short reads and long-range data were obtained from a *T. molitor* male pupa. Sequencing transcripts from 12 *T. molitor* life stages resulted in 279 million reads for gene prediction and genetic engineering. A unique plasmid delivery system containing guide RNAs targeting the eye color gene *vermilion* flanking the muscle *actin* gene promoter and EGFP marker was used in CRISPR/Cas9 transformation. Results: The assembly is approximately 53% of the genome size of 756.8 ± 9.6 Mb, measured using flow cytometry. Assembly was complicated by a satellitome of at least 11 highly conserved satDNAs occupying 28% of the genome. The injection of the plasmid into embryos resulted in knock-out of *Tm vermilion* and knock-in of EGFP. Conclusions: The genome of *T. molitor* is longer than current assemblies (including ours) due to a substantial amount (26.5%) of only one highly abundant satellite DNA sequence. Genetic sequences and transformation tools for an insect important to the food and feed industries will promote the sustainable utilization of mealworms and other farmed insects.

## 1. Introduction

The yellow mealworm, *Tenebrio molitor* (Coleoptera: Tenebrionidae), is a cosmopolitan pest of mainly poultry farms. However, *T. molitor* also represents a group of insects that are being incorporated as a source of alternative protein in food and feed for a burgeoning world population [1]. We are interested in the genetics of insect species farmed for food, feed, and other applications [2,3].

Mealworms have been reared commercially mostly as pet food for reptiles and birds. However, in the past decade a new industry has emerged to leverage the significant environmental benefits of farming insects compared with other livestock [4]. These insects represent a high-protein, low-fat food that is also rich in vitamins and minerals [5]. In addition, mealworms have a lower environmental impact than traditional livestock, requiring less land, water, and input feed to produce the same amount of protein as traditional livestock [6]. Rearing insects for food applications in an indoor closed system can reduce emissions and prevent disease outbreaks in colonies [7]. Insect farms are increasingly rearing insects vertically, further reducing their footprint and land requirements, and near food manufacturing facilities to minimize transportation needs.

Insects, particularly *T. molitor*, have gained attention as a sustainable and nutritious food source for humans and animals, especially in regions facing food insecurity [8]. While the USA primarily utilizes farmed insects as supplemental feed for animals, particularly poultry and fish, there is growing recognition of the potential of insects as an alternative to traditional meat production, which significantly contributes to climate change [7]. The European Food Safety Authority’s approval of *T. molitor* for human consumption underscores the potential for edible insects to serve as future food and feed due to their beneficial nutritional characteristics and low environmental impact [9]. Furthermore, *T. molitor* larvae are recognized for their high nutritive value, including high lipid and protein content, making them a valuable food source [10]. The economic significance of *T. molitor* in large-scale conversion of plant biomass into protein further highlights its potential as a sustainable food and feed source [8].

Insects are relatively easy to breed and maintain in the laboratory and have been used as model organisms for studying a variety of biological questions. Insects are an important part of the food chain, and they play a vital role in pollination and other critical ecological services [11,12,13,14,15]. Insects can also be used as biofactories for various applications, including protein production and biomanufacturing [4]. Insect-based biomanufacturing efforts have led to the development of bioreactor systems and low-cost media formulations, enhancing the efficiency and cost-effectiveness of recombinant protein production [16]. Bioconversion technology using insects provides opportunities to produce feed and energy from by-products of the agro-food industry [17].

Scientists are incorporating insects into the food chain as part of a circular economy that will reduce the environmental impact of mealworm production, such as developing more efficient feeding methods including robotics, or incorporating recycled waste products [18,19,20,21]. We and others are trying to improve the nutritional value of mealworms through diet experimentation or genetic manipulation. Ideally, improvements such as increased protein content and other value-added research will make mealworms a more appealing food source for humans and other animals.

The genomes of insects contain the genes and regulatory elements that contribute to their development and biological function, determining the genetic basis for specific traits, such as resistance to pesticides or the ability to tolerate extreme environments. Sequencing insect genomes is also a powerful tool to understand evolutionary relationships, to identify genes that have been conserved over time, and to develop new model organisms. Recently, genome assemblies for *T. molitor* have been released [22,23,24]. In this paper, we compare our assembly metrics with published long-read assemblies, provide an analysis of transcripts from *T. molitor* life stages, further explain how the relatively unique and highly conserved satellites contribute to the evolution of insects, and describe technology that can harness the genetic information into applications for agriculture. Our data indicate that the genome is larger than previous measurements, and the importance of the genome assembly for the insect food industry is a major step in the development of insects for downstream food applications.

## 2. Materials and Methods

### 2.1. Insects

All samples originated from a laboratory colony of *T. molitor* reared at the Center for Grain and Animal Health Research (CGAHR), Manhattan, KS, USA, at 27.5 °C, 65% R.H., 0L:24D on a mealworm diet of 50% rolled oats, 2.5% brewer’s yeast, and 47.5% wheat flour.

For genetic engineering, *T. molitor* were received from CGAHR at All Things Bugs LLC in 2018 and maintained as a lab colony for multiple generations. Larvae were reared in cages consisting of a shoe box (6-Quart, Sterilite, Townsend, MA, USA) with a 150 × 30 mm opening on the lid covered by screen mesh material. Larvae were fed wheat bran in the lower third of the box (Nuts.com, Cranford, NJ, USA) with 10 mL of hydride water crystal (Prestige Import Group, Deerfield Beach, FL, USA) in a Petri dish (150 × 15 mm, VWR, Radnor, PA, USA) placed on top of the wheat bran, with 3–5 mL water crystals provided biweekly. Pupae were transferred by hand to a separate box with some wheat bran but no water crystals until they emerged as adults. Adults were removed twice a week to another adult egg-lay box containing sifted wheat flour (Nuts.com, Cranford, NJ, USA), with tissue paper over the flour to hold the water crystal dish. Twice weekly, adults were removed using a noodle scoop (356 × 155 mm, Goodcook, Broadway, CA, USA) to an empty box, and flour was sifted using a sieve (12′ No. 25 mesh, Advantech, Danvers, OH, USA) to collect eggs, with a small paint brush used to remove any eggs on the cage walls. Eggs were placed in a Petri dish (150 × 10 mm) until hatched and adults replaced on the sifted flour.

### 2.2. Genome Sequencing and Assembly

Male and female *T. molitor* adults were shipped to Texas A&M University, College Station, TX for flow cell cytometry analysis of the genome size of *T. molitor*, performed as described in [3,25]. Genomic DNA was extracted from a single male *T. molitor* pupa using a previously described protocol [26] and an E.Z.N.A. Insect DNA Kit (Omega BioTek, Norcross, GA, USA). Approximately 30 micrograms of gDNA with an average size of >50 kb was hand delivered to the sequencing facility at the U.S. Meat Animal Research Center, Clay Center, NE, USA. Size selection of a portion of the gDNA was via BluePippin (Sage Science Inc., Beverly, MA, USA) using a 15 kb lower cutoff value. Libraries for long-read sequencing on the RSII platform were constructed using the SMRTbell™Template Prep Kit 1.0 as recommended by the manufacturer (Pacific Biosciences, Menlo Park, CA, USA). Four libraries were prepared and sequenced on sixteen SMRT cells of the PacBio RSII using P6/C4 chemistry (eight cells each) to 44× coverage.

Short-read sequencing was with gDNA extracted from male and female *T. molitor* pupae. Each insect was an individual biological replicate with variable technical replicates as indicated: i.e., four male pupae = four biological and 12 technical replicates; two female pupae, five technical replicates; and male and female adults as above: seven male adults, 11 technical replicates; two female adults, five technical replicates. Libraries were made with a Nextera DNA Flex kit on a NeoPrep and were sequenced on MiSeq 2 × 300 paired-end (all Illumina products, San Diego, CA, USA). Sequences were submitted to NCBI SRA accession # SUB13823713.

The assembly of PacBio reads from *T. molitor* was undertaken using a customized CANU [27] script to suppress repeats, because running time under default parameters was estimated at 20 k CPU hours (similar to the human genome) and was overwhelming data storage. The assembly was run with Canu v1.3 + 148 commits (r7764) with an added option corMhapFilterThreshold = 0.0000002. Additionally, within the MHAP overlapper the repeat-idf-scale was set to 50 rather than the default of 3. The first parameter marks more k-mers as repetitive from the full list; the second makes the overlapper more strongly prefer the non-repetitive k-mers. After the final assembly, any output in asm.unassembled.fasta that was composed of one more than one read was added to the final assembly sequences. Post-assembly processing included arrow polishing and a screen through purge_haplotigs [28].

A different *T. molitor* male pupa was shipped to Cantata (formerly Dovetail, Scotts Valley, CA, USA) for scaffolding. Scaffolding details for Chicago and Hi-C using HiRise was as previously described [29,30]. A hybrid assembly was obtained by using the Chicago/HiRise scaffolds in a reference-guided assembly with short reads (see above) in SeqMan NGen 14.1.0 build 115 (DNAStar, Madison, WI, USA), and that hybrid assembly was used by Cantata for the final scaffolding with Hi-C data (also in HiRise). The completeness of coding sequences in each assembly was evaluated by [31,32] using insecta_odb10 (Creation date: 10 September 2020). The scaffolded assembly was submitted to NCBI with accession # JAWJDX010000000.

### 2.3. Gene Prediction, Annotation, and Analysis

Genes in the *T. molitor* genome assembly were predicted in Omicsbox 3.0.30 [33] (Valencia, Spain) in the package Augustus 3.4.0 [34] set to eukaryotic gene finding, using *T. castaneum* as the closest species and life stage/sex transcripts as extrinsic evidence data. Translated proteins were submitted to CloudBlast using blastp against reference proteins (ref-seq_protein v5) and a taxonomy filter 7070 (*T. castaneum*). Gene Ontology (GO) annotation mapping [33] was complemented by Interproscan [35]. Enrichment analyses were performed using ShinyGO v0.77 (FDR 0.05) [36] and Panther 17.0 [37,38].

Synteny analyses were performed on an HPC cluster (CERES, ARS USDA) using BUSCO output for our assembly (Tmo) against *T. castaneum* assembly 5.2 (Tcas5.2, accession # GCA_000002335), as well as also comparing another *T. molitor* assembly (Tmo_v3, accession # GCA_907166875.3 [23] to Tcas5.2. We used BUSCO software, version 5.4.7 [32] with their lineage dataset insecta_odb10, with 75 genomes and 1367 BUSCOs. BUSCO data that matched scaffolds from Tmo or Tmo v3 were used only when the scaffold’s size was greater than 10 million base pairs. RIdeogram v.0.2.2 [39] was used to create the genome synteny graphs showing Tcas 5.2 versus Tmo or Tmo v3. SQLite [40], software that provides a relational database platform, was used to reformat the data to conform to RIdeogram’s input requirements. The modified BUSCO output full_table.tsv files from the new Tmo scaffolds run and the Tmo v 3 BUSCO run were inputs to SQLite. These modified tables were joined on the BUSCO ID using SQLite. The resulting table provided the data RIdeogram used to create the Bézier curves from the top horizontal bars to the bottom horizontal bars. RIdeogram also requires the length of each chromosome for each of the assemblies. One hurdle arose while attempting to use RIdeogram with horizontal bars not labeled with sequential ordinal numbers starting with one. A user can input non-ordinal labels, but the Bézier curve data must contain ordinal numbers. To overcome this hurdle, an additional SQLite table was used to match our horizontal bar labels with the required ordinal numbers.

### 2.4. Transcriptome Sequencing and Analysis

Twelve *T. molitor* life stages were sampled, including eggs (eggs), early larvae (el), middle larvae (ml), late larvae (ll), early female pupae (efp), early male pupae (emp), late female pupae (lfp), late male pupae (lmp), early female adult (efa), early male adult (ema), late female adult (lfa), and late male adult (lma) (Table S1). Life stages were stored in Zymo DNA/RNA shield at −20 °C and extracted using a Zymo Insect RNA kit (both products of ZymoResearch, Irvine, CA, USA), and libraries were made using Corall polyA selection and total RNA library prep kits (Lexogen, Vienna, Austria). Libraries were sequenced on a NextSeq P2 200 cycle with 101 × 101 bp paired-end reads (Illumina). Samples included 3–4 biological replicates and total reads per life stage and ranged from 20–30 million reads, except for late male pupae with only 2 replicates and 7 million reads (Table S1). In all, a total of 279 million reads were used in the identification of genes in the genome assembly as well as to identify sequences used in CRISPR Cas9 targeting. Reads were submitted to NCBI SRA accession # PRJNA1012330.

Reads were aligned to the *T. molitor* genome assembly in ArrayStar v. 17 (DNAStar Lasergene, Madison, WI, USA). Annotations were uploaded, and gene names, descriptions, RPKM values, and GO terms were collected in a spreadsheet (File S1). Genes similar to *T. castaneum* were used in enrichment analyses. The differential expressions of all genes in all life stages were compared (ANOVA, *p* < 0.05), and those genes below the statistical cutoff were analyzed by ShinyGO [36]. Genes with expression levels > 1000 RPKM were input into Panther (v 17.0; [37]) for functional analysis of the protein class against the *T. castaneum* genome.

### 2.5. Satellite DNA (SatDNA) Detection and Analysis

Using the tools for processing FASTQ data at the Galaxy server (https://repeatexplorer-elixir.cerit-sc.cz/galaxy/, accessed on 1 March 2023), MiSeq reads from *T. molitor* female adult specimens were pre-processed, quality-filtered, and trimmed. Low genome coverage (<0.50×) is optimal for the identification of repetitive sequences by RepeatExplorer2 and TAREAN computational tools [41], as reads that originate from repetitive elements produce multiple similarity hits. Thus, they can be identified as clusters of frequently appearing sequences, while almost no similarities are detected between reads derived from single-copy sequences. The reads were randomly subsampled to obtain sets that were within the optimal genome coverage range. Similarity-based read clustering was performed on the Galaxy server under the default parameters, using several randomly subsampled sets, which contained 35,208, 31,453, and 262,363 reads, corresponding to a genome coverage of 0.006×, 0.007×, and 0.05×, respectively. Upon clustering, consensus sequences of satDNAs were obtained in the TAREAN report. The number of reads in each cluster is proportional to the genomic abundance of the corresponding satDNA, making this an assembly-independent method of choice in satDNA detection and abundance determination [41]. Consensus sequences of satDNAs obtained via three rounds of read clustering were compared to each other using discontinuous megablast with the default parameters in Geneious Prime (Biomatters Ltd., Auckland, New Zealand) to detect clusters belonging to the same satDNA from different analyses and all subsequent sequence analysis. The abundance of the satDNAs comprising the satellitome was averaged from the outputs of the three clustering analyses. Consensus sequences of the 11 satDNAs constituting the satellitome of *T. molitor* are in File S2. These were used for annotation of satDNAs in *T. molitor* scaffolds, allowing 70% divergence to the consensus, to include different monomer variants. Employing NCBI BLAST (accessed August 2023), a blastn search was performed on whole-genome shotgun contigs (WGS) and the nucleotide collection (nt) of insect databases, using the consensus sequences of the 11 satDNAs. For multiple matches belonging to the same species, the best one with respect to the query coverage and identity score was presented. The separation time of insect orders was generated as a “Timetree” in the TimeTree of Life database [42] (http://www.timetree.org/ accessed on 1 February 2023).

### 2.6. CRISPR

#### 2.6.1. Design of the CRISPR Target Gene

A total of 120 bp of the first and second exon of the *Tm vermilion* gene was chosen as the CRISPR target site, within which three CRISPR targeting sites were used to design three different sgRNAs for knock-out (GGP sgRNA Designer, Broad Institute) experiments (Table S2). The target sgRNA sequences were from Synthego (Redwood City, CA, USA). To verify the knock-out sequence in *T. molitor*, gDNA was extracted using a Quick-DNA insect miniprep kit (ZymoResearch). PCR using a forward primer “5′-CGTAAACACTCGACTTCC-3′” and reverse primer “5′-ATCAACACGACACGATTCAG-3′” was used to amplify the target sequence from gDNA. The PCR product was isolated and eluted on a 1% agarose gel, and a sequencing primer “5′-TACGTCAGCTTGATTAAGTC-3′” was used to verify the knock-out (Oklahoma Medical Research Foundation DNA Sequencing Facility, Oklahoma City, OK, USA).

#### 2.6.2. Plasmid DNA Construct Design

The marker gene enhanced green fluorescent protein (EGPF) driven by the *Tm muscle actin* gene promoter was used as the knock-in construct via CRISPR/Cas9. The muscle actin gene from *T. castaneum* was used in a reciprocal BLAST search to identify the *T. molitor* ortholog from the transcriptome data, and the mRNA sequence from that search was used to identify the *Tm muscle actin* gene in the genome assembly. To identify the promoter for the *Tm muscle actin* gene, an upstream DNA sequence 1 kb from the start codon was submitted to Neural Network Promoter Prediction (Berkeley Drosophila Genome Project, https://fruitfly.org/seq_tools/promoter.html). The 732 bp predicted promoter sequence and 5′ UTR for *Tm muscle actin* was placed upstream of the EGFP coding sequence as the marker gene and an sv40 polyadenylation sequence. CRISPR knock-in sites consisted of 121 bp of gDNA on either side of the marker gene for the final knock-in DNA construct, Tm-actin-EGFP-KI. The construct was synthesized using GeneScript (Piscataway, NJ, USA) in a pUC57 vector plasmid as the final product.

#### 2.6.3. Microinjection

The *T. molitor* microinjection process was similar to that used in a previous study [43]. Components of the knock-in microinjection solutions were: 100 ng/mL of DNA construct Tm-actin-EGFP-KI, 6 pmol/mL of sgRNA or 18 pmol/mL total of all three sgRNA together, 1 µg/mL of TrueCut Cas9 protein V2 (ThermoFisher, Waltham, MA, USA), and 20% phenol red buffer (Sigma Aldrich, St. Louis, MO, USA). The solution was mixed and incubated at room temperature for 5–10 min for Cas9-sgRNA binding and was kept on ice during microinjection. Either three sgRNAs combined or a single sgRNA (#1, #2, or #3) were injected (Table S2). In addition, two sets of microinjections with either 100 ng/mL of DNA construct Tm-actin-EGFP-KI or 100 ng/mL of DNA construct Tm-actin-EGFP-KI with 1 µg/mL of TrueCut Cas9 protein V2 (both sets without sgRNA) were injected as negative controls.

#### 2.6.4. Screening for CRISPR Knock-Out/in and Established Colonies

To screen for either white eye (*Tm vermilion* knock-out) or EGFP (EGFP knock-in) expression phenotypes, freshly hatched G_0_ *T. molitor* larvae were observed under a dissection microscope with standard LED white-light to look for eye color disruption, or a fluorescent dissecting microscope (Leica M125, Leica Microsystems Inc., Deerfield, IL, USA) with a blue fluorescent light and GFP filter to identify knock-in EGFP expression phenotypes. G_0_ larvae with a positive eye color knock-out phenotype but no EGFP detection were reared to pupae as “knock-out” larvae in a 16 oz deli container (S-24414, Uline, Pleasant Prairie, WI, USA) with wheat bran and a ventilated lid. G_0_ larvae with the EGFP-positive phenotype were reared to pupae in separate containers as “knock-in” larvae. G_0_ larvae with the wild-type eye phenotype and no EGFP expression were not retained. As “knock-out” larvae became pupae, they were separated by sex into two new pupae containers for eclosion. Adult “knock-out” self-crosses (two males and four to six females from the same injection group) were transferred to a 16 oz deli container filled with sifted flour, covered by tissue paper to retain a small Petri dish lid (35 × 10 mm, VWR, PA, USA) with water crystals, and covered by a ventilated lid. A sieve (No. 25 mesh) was used to sift out flour and collect eggs weekly. Eggs from crosses from each treatment group were transferred to separate Petri dishes, and newly emerged larvae were screened for both knock-in and knock-out phenotypes. Knock-in pupae were separated by sex into male/female pupae containers until adults. Small out-cross groups were set up using EGFP-positive G_0_s with one G_0_ out-cross to 2–3 wild- type beetles, labeled “knock-in” out-crosses. Eggs from out-cross groups were collected as described above. G_1_ generations were screened, and all white eye color phenotypes were selected as knock-out G_1s_, and EGFP positives were selected as knock-in G_1s_. For all G_1_s without white eyes or EGFP expression from knock-in crosses, one additional self-cross was screened to recover the white eye or EGFP phenotype (referred to as G_1_ self-crosses).

## 3. Results

In the following sections, we provide metrics for our genome assembly of *T. molitor*, compare those metrics to those of other long-read assemblies, briefly describe transcripts from different *T. molitor* life stages, characterize and further analyze the satellitome in *T. molitor*, and describe a plasmid system for both simultaneous knock-in and knock-out of genes.

### 3.1. Results

#### 3.1.1. Genome Assembly of *T. molitor*

We assembled the genome sequences from short- and long-read data, as well as long-range data, as follows. A modified CANU script was used to assemble the PacBio long reads into a draft assembly. The metrics of the draft assembly were 417,676,750 bases total length, 7484 contigs, N_50_ of 103,701 bases; the BUSCO score was 96.1% C, 82.7 single, 13.4 duplicate, 1.2 fragment, 2.7 missing (Table 1).

The draft assembly was input into Chicago/HiRise scaffolding, which significantly reduced the number of contigs to 2364 and increased the N_50_ to 2.01 Mb as well as the size of the assembly to 423,052,750 bases. At this point, we used the Chicago/HiRise scaffold in a reference-guided assembly with short reads (see above) to try to improve both contiguity and base-call accuracy, resulting in a hybrid assembly with a reduction of contigs to 1293 and total of 400,747,566 bases and slight improvement of N_50_ to 2.12 Mb. The hybrid assembly was then used as the input for scaffolding with Hi-C data in HiRise, reducing the number of scaffolds further to 1031, and total bases of 400,765,366, but a marked improvement in N_50_ to 26.2 Mb. BUSCO scores also gradually increased to a final score of 98.3% in the final assembly (C: 98.3% [S: 83.2%, D: 15.1%], F: 0.5%, M: 1.2%, n: 1367). While the scores indicated that most reference genes were found in the assembly, there were also 15.1% duplications, likely due to the difficulty in purging duplications from the draft assembly.

**Figure 1:**
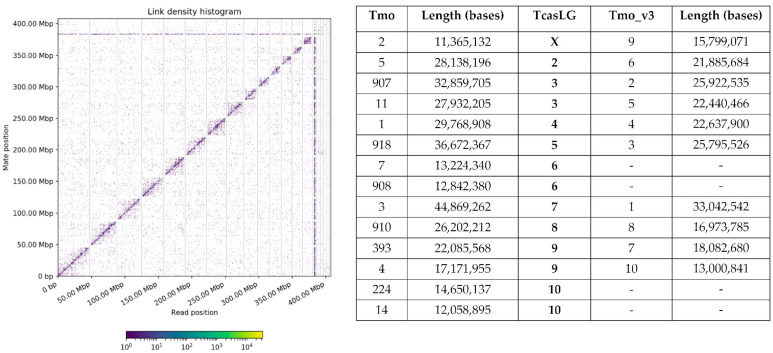
**Link density histogram (left) and table of scaffolds from our *T. molitor* assembly (right).** On the right, comparison of our assembly (this study, Tmo), *T. castaneum* Tcas5.2 (TcasLG), and a previous *T. molitor* assembly [23] (Tmo_v3). Correspondence of scaffolds to the LG of Tcas5.2 was made through synteny analysis of GCA_907166875.3 and *T. castaneum* 5.2, as well as our assembly (Tmo) vs. Tcas5.2, visualized from the results of the BUSCO analyses in each genome assembly (Figure S1).

The Hi-C histogram indicated that there were 14 large scaffolds approximating chromosomes (Figure 1). The scaffolds ranged from 11.4 to 44.9 Mb and corresponded to the linkage groups (chromosomes) in *T. castaneum* through a synteny plot of the BUSCO genes in the two genomes (Figure S1). There were 1454/1575 BUSCO genes found in the larger scaffolds (>10 Mb) of our *T. molitor* genome assembly, whereas the BUSCO analysis in another genome assembly (Tmo_v3) [23] identified 1191/1575 genes in their larger scaffolds. Visualization of BUSCO genes mapping to the LG of Tcas5.2 and either our assembly (Tmo) or GCA_907166875.3 (Tmo_v3) identified fragmented and missing BUSCO genes (Figure S1). Four scaffolds in our assembly (Tmo) were fragmented: scaffolds 907 and 11 corresponded to Tcas5.2 linkage groups (TcasLG) 3; 7 and 908 corresponded to LG6; 393 and 4 corresponded to LG9; and 224 and 14 corresponded to LG10. Tmo_v3 scaffold 1 corresponded to Tcas5.2 LGX and was larger than our corresponding scaffold 2. However, the remaining Tmo_v3 scaffolds were smaller than our Tmo scaffolds, and there were no BUSCO genes from Tcas LG6 or 10 found in the larger scaffolds of Tmo_v3.

Augustus gene prediction found 39,608 genes among 944 scaffolds in our genome assembly (File S1). However, 81.2% of the genes were found in the 14 larger scaffolds (>10 Mb). Predicted proteins were submitted to blastp against the *T. castaneum* database for downstream analysis. Annotation of the predicted genes indicated that approximately 47% of the annotations either had no annotation, or were hypothetical/unnamed/uncharacterized.

The genome size for *T. molitor* was estimated using flow cytometry. Female *T. molitor* were estimated to have a 791.6 ± 9.3 Mb (n = 4) genome, whereas for males it was 756.8 ± 9.6 Mb (n = 2). These values are considerably higher than previously reported in other *T. molitor* genome assemblies [23,24,25], including ours.

#### 3.1.2. Comparison of *T. molitor* Genome Assemblies

We compared our genome assembly of *T. molitor* to those that also used long-read sequencing (Table 2). Our genome assembly (Tmo) was about 53% of the genome size that was predicted by our flow cytometry analysis, compared to 33–38% in the other two assemblies (GCA_907166875.3; GCA_027725215.1). We extracted gDNA for a single male pupa, as did GCA_907166875.3, whereas GCA_027725215.1 extracted gDNA from the head and legs. We were the only long-read assembly using PacBio (RSII P6), whereas the other two relied on Oxford Nanopore technology. All three assemblies incorporated Illumina short reads into the assemblies. The predicted number of genes was higher in our Tmo assembly (35,281) than the other assemblies (19,830 and 21,418), likely due to duplication in our scaffolds due to difficulties in purging haplotigs. The total number of scaffolds in our Tmo assembly (1031) was intermediate between GCA_907166875.3 (111) and GCA_027725215.1 (1986), but the scaffold N_50_ for our assembly (26.2 Mb) was longer than the other assemblies (20.8 and 21.9), and the scaffold L_50_ was 7 compared to 6 for the other assemblies. We used both Chicago and Hi-C scaffolding, whereas GCA_907166875.3 used only Hi-C (both by Dovetail/Cantata), and we didn’t find evidence of scaffolding with GCA_027725215.1.

### 3.2. Satellites in T. molitor

To obtain an overview of the complete inventory of satDNAs in the genome of the *T. molitor*, several rounds of TAREAN clustering were performed on three subsampled sets of short reads. The combined results of the three analyses resulted in 11 satDNA which constituted the satellitome of this species and occupied 28% of the genome (Table 3). The detected satDNAs presented a range of monomer lengths, from 93 (TmSat11) to 735 bp (TmSat8). TmSat1 was most abundant, constituting 26.5% of the *T. molitor* genome, and corresponded to the already described 142 bp repeat known to make up a significant part of the yellow mealworm genome [44]. Genome occupancy of 10 novel satDNAs comprising the satellitome was relatively low, ranging from 0.45% (TmSat2) to 0.01% of the genome (TmSat11).

The distribution of the 11 satDNAs was inspected using an in silico analysis on *T. molitor* scaffolds by annotating consensus sequences of each satDNA on the novel genome assembly. The number of monomers detected, number of scaffolds occupied, monomer identity, and assembly occupancy for each satDNA is presented in Table 3. The major satDNA of this species, TmSat1, occupies the largest number of scaffolds; however, one stands out (TmoDt5_rgH0X_scaff_79) as being replete with this sequence. In fact, that phenomenon actually can be visualized in the link density histogram in the horizontal and vertical lines near 375 Mbp (Figure 1). Monomers of all satDNA in this species present notable sequence preservation, with average monomer identity from 83.8 (for TmSat9) to 98.6% (for TmSat3). Eleven satDNAs occupy 0.79% of the assembly, with 0.41% belonging to the TmSat1.

**Figure 2:**
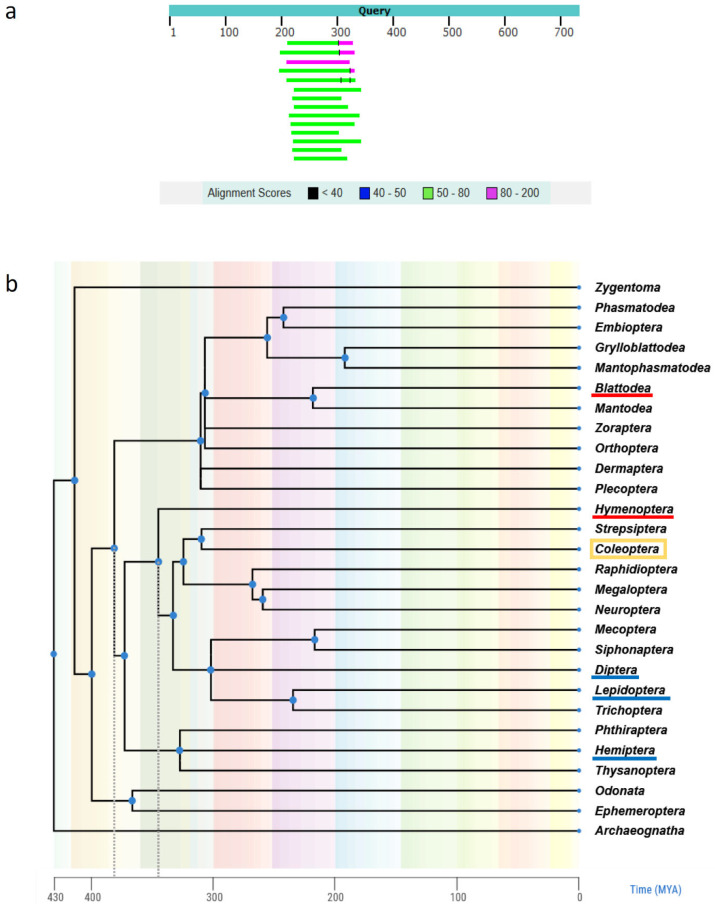
**TmSat8 found in insect orders.** (**a**) Location of similarity hits belonging to *Zophobas atratus*, *Tribolium madens*, *T. freemani* and *Latheticus oryzae* with respect to the TmSat8 query sequence. (**b**) Timetree presenting separation time of insect orders. *T. molitor* order (Coleoptera) is marked yellow, orders of species which show similarities that encompass almost 100% of satDNA monomer length while keeping high sequence identity are underlined in red, and orders of species with similarities restricted to satDNA monomer segments are underlined in blue. Dashed gray lines denote Coleoptera-Blattoidea and Coleoptera-Hymenoptera separation times, denoting minimal age of TmSat1 (380 MYA) and TmSat7 (340 MYA).

The satDNA sequences were subjected to BLAST analyses in a search for similarity with publicly available sequences deposited in NCBI GenBank databases. The search revealed similarity in 9 out of 11 inspected satDNAs (except TmSat9 and TmSat11) that were found in other insect genomes (Table S3). Few species show similarity that encompasses almost 100% of monomer length, while keeping high sequence identity (TmSat1 in *Solenopsis invicta*, *Periplaneta americana*, *Zophobas morio* and *Tenebrio obscurus*, and TmSat7 in *Dinoponera quadriceps*). Only *T. obscurus* and *Z. morio* belong to the same order as *T. molitor* (Coleoptera), while three other species belong to orders Blattoidea (*P. americana*) and Hymenoptera (*S. invicta* and *D. quadriceps*). In other species, similarity to monomer segments was found, varying in size (Table S3). However, even short similarity stretches from different species are frequently situated in the same segment of the monomer sequence (exemplified in Figure 2a), indicating the potential existence of a conserved box within the satDNA sequence. Insect species showing similarity to satDNA sequences of *T. molitor* belong to the orders Coleoptera (17), Lepidoptera (13), Hymenoptera (4), Diptera (6), Hemiptera (2), and Blattodea (1) (Table S3). Timetree indicated the time of separation of insect orders (Figure 2b), in which Coleoptera and Blattoidea have separated ~380 MYA, and Coleoptera and Hymenoptera ~340 MYA. The presence of TmSat1 in species from the orders Coleoptera and Blattoidea indicates that Sat1 is derived from the common ancestor, with the minimal estimated age of this satDNA sequence as 380 MY, and a minimum age of TmSat7 being approximately 340 MY. Two satDNAs, TmSat9 and TmSat11, have not been detected in any of the inspected insect species (with currently available genomic data), which suggests that these satDNA sequences arose in the genome of *T. molitor* later in the evolutionary history.

### 3.3. Transcripts from T. molitor Life Stages

Transcripts were sequenced from 12 different life stages/sexes of *T. molitor.* These transcripts were used for the prediction of genes in the genome assembly and annotation of these genes. We briefly examined the enrichment of GO terms in significantly differentially expressed genes and those that were most highly expressed among the samples.

There were 3078 genes that were differentially expressed among all life stages (*p* < 0.05). The most highly enriched GO term in that group was glycosyl hydrolases family 18, whereas the term with the lowest FDR and the largest number of genes was hydrolase (Figure S2). These terms are likely reflective of the large shifts in genes associated with feeding and metabolism occurring in different life stages or between male and female. Other terms included cytochrome P450, in particular those from class E, group I, including CYP1 and CYP2 which mainly detoxify, and CYP17 and CYP21 which metabolize endogenous compounds.

**Figure 3:**
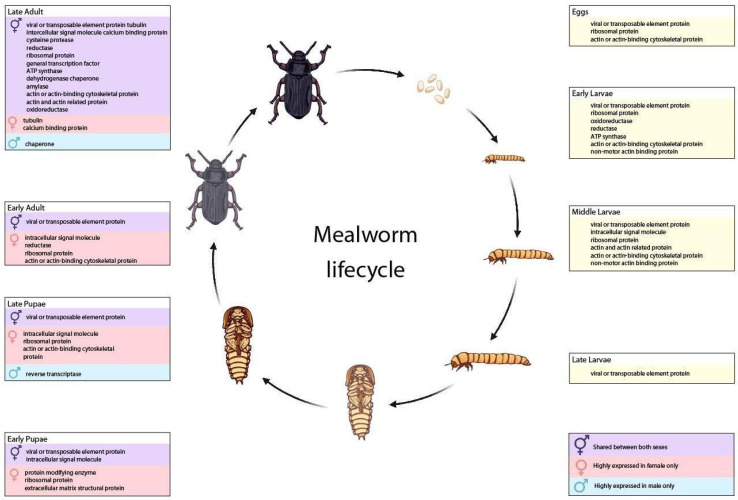
**Enrichment of GO terms in transcripts from *T. molitor* life stages or sexes.** Enriched GO terms in transcripts from eggs to adults, and male/female pupae and adults, were associated with life processes at each stage.

The number of genes expressed > 1000 RPKM in each life stage/sex was similar (Table S4). In five of the 12 life stages/sexes, the highest expressed gene was g38548.t1 which encodes a predicted 67 aa hypothetical peptide that is highly conserved in other insects but also other organisms including bacteria and fungi. Using an enrichment of gene ontology (GO) terms in the most highly expressed genes, we found that the term “viral or transposable element protein” was shared among every sample (Figure 3). The male and female late-adult stages contained more genes with defined annotations than other stages. The early stages of *T. molitor* focus on growth and development (and enriched GO terms including oxidoreductases, ribosomal subunits, and cytoskeletal development). In adults, in addition to GO terms associated with continued development and reproduction, we also found more genes encoding dietary enzymes such as amylase or cysteine peptidases.

### 3.4. Genetic Engineering

To disrupt *vermilion* gene expression in *T. molitor*, 2759 eggs were microinjected with four different sgRNAs (#1, 2, 3, or 1–3) and 1272 larvae (36% to 57%) were successfully hatched, with an average hatch rate of 46% (Table S5). CRISPR efficiency was evaluated in microinjections with individual injections of sgRNA #1, 2, or 3 or a combination of #1/2/3 (Table S2). Complete knock-outs were easily identified in larvae; however, partial knock-out or mosaic phenotypes were difficult to distinguish since the eye spots in *T. molitor* larvae are very small. Thus, we did not follow the knock-out rates in G_0_. However, all sgRNA injections resulted in some larvae with complete knock-out phenotypes (i.e., loss of eye color), and those individuals were used for the knock-out self-crosses. Eye color was not disrupted in negative control treatments.

**Figure 4:**
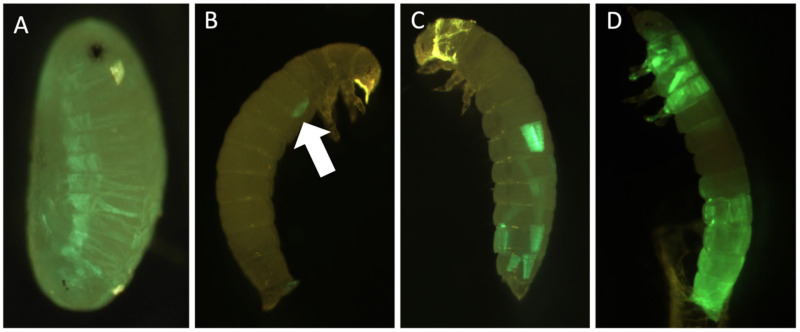
**EGFP transient expression in *T. molitor* after microinjection.** (**A**) Developed embryo, (**B**) G_0_ larva with low EGFP transient expression (white arrow), (**C**) G_0_ larva with 10% of muscle with EGFP transient expression, (**D**) G_0_ larva with 40% of muscle with EGFP transient expression.

The G_0_ EGFP transient expression rate was very low when using three sgRNAs (13%) compared with just one (50–69%) (Table S5). However, the EGFP transient expression in G_0_ was observed as early as 3–4 day-old eggs in the classic striated muscle fiber structures fluorescing in green under blue light (Figure 4). The localization and levels of expression varied between different individuals in all injection groups (Figure 4B–D). EGFP transient expression was detected in all treatments including those without sgRNAs, and phenotype was the same as EGFP expression in the muscle tissue.

**Figure 5:**
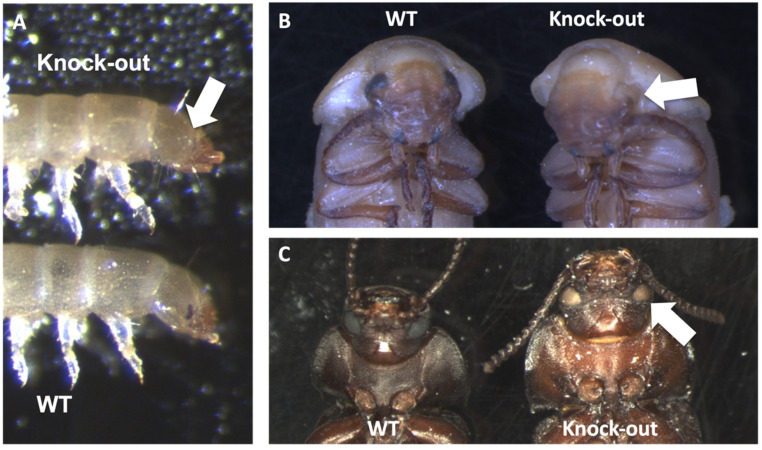
***Tm vermilion* knock-out phenotype in different life stages.** Knock-out phenotype is denoted by the white arrows. (**A**): Larva, (**B**): Late pupa, (**C**): Adult.

All injections with sgRNA provided G_1/2_ individuals with the knock-out phenotype (Table S6). The successful knock-out phenotype was a reduction of black eye pigmentation to a light brown color in pupae and adults, and a complete loss of pigmentation in young larvae (Figure 5). The knock-out rate was from 30% to 47% with injection of individual sgRNA, and sgRNA #1 had the highest number of knock-outs compared to other individually-injected sgRNAs. However, the highest knock-out rate, 54%, was from all three sgRNAs injected together.

There was one out-cross and two groups of self-crosses following the examination of G_0_ and G_1_ insects. We first out-crossed G_0_ GFP-positive individuals with wild- type insects. The self-crosses were set up as G_0_ sgRNA-injected individuals with an altered phenotype, and another group from G_1_ insects lacking EGFP-expression but with possible knock-outs of *Tm vermilion*. The progeny for the potential G_1_ eye-color knock-out cross were again crossed and eye color was again evaluated in G_2_. All G_0_ injected with sgRNA resulted in some individuals with a G_1_ knock-out phenotype (Table S6). Knock-out self-crosses had much lower knock-out rates (4 out of 25 crosses, 16%) compared to G_1_ self-crosses (22 out of 36 crosses, 61%). In two sgRNA groups, we were not able to detect any G_1_ knock-out individuals in the self-crosses, but all single sgRNA injection groups had G_1_ self-crosses with knock-out individuals.

**Figure 6:**
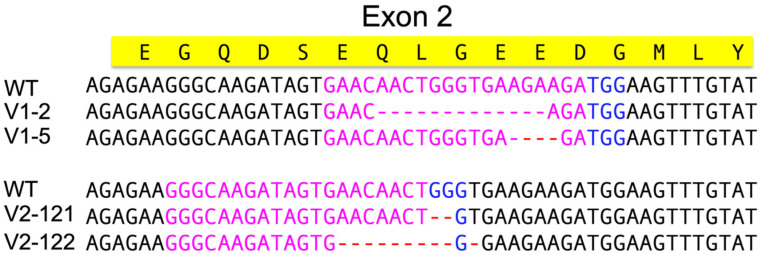
**Sequencing result of *Tm vermilion* knock-out in G_1_ individuals.** Different deletions were detected in the sgRNA target sequences close to the PAM in different knock-out events. Pink color sequences are the sgRNA target sequences. Blue color sequences are PAMs. Red color hyphens are the sequence deletions. V1: G_1_ individuals from sgRNA#1 injection groups. V2: G_1_ individuals from sgRNA#2 injection groups.

Sequencing of the target gene *Tm vermilion* from injection groups sgRNA #1 and #2 was used to evaluate CRISPR effects. The sequencing result indicated indels around the sgRNA targeting area (Figure 6).

**Figure 7:**
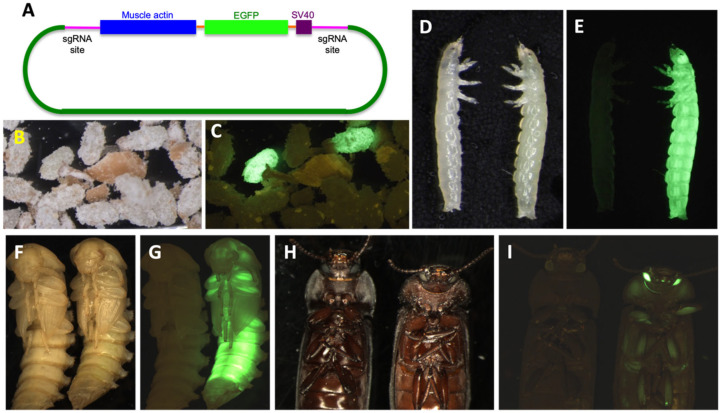
**EGFP knock-in phenotype in *T. molitor*.** (**A**): Knock-in construct design. (**B**): EGFP-positive and wild-type eggs under white light. (**C**): EGFP-positive and wild-type eggs under fluorescent screening. (**D**): Wild-type (left) and EGFP-positive (right) larvae under white light. (**E**): Wild-type (left) and EGFP-positive (right) larvae under fluorescent screening. (**F**): Wild-type (left) and EGFP-positive (right) pupae under white light. (**G**): Wild-type (left) and EGFP-positive (right) pupae under fluorescent screening. (**H**): Wild-type (left) and EGFP-positive (right) adults under white light. (**I**): Wild-type (left) and EGFP-positive (right) adults under fluorescent screening.

The EGFP knock-in was not detected in G_1_ individuals from the combination #1/2/3 sgRNAs injection. However, EGFP-positive individuals were observed in G_1_ individuals from each of the single sgRNA injections. sgRNA #2 had the highest knock-in rate, 30%, and the lowest was in the sgRNA #1 group, 10% (Table S7), with the muscle EGFP expression phenotype observed in different life stages (Figure 7). Contrary to the combination #1/2/3 EGFP knock-in rate, the knock-out rate for the triple sgRNA injection was the highest (53%) (Table S6). The knock-out rate for individual sgRNA injections ranged from 30% to 47%, with the highest knock-out rate with sgRNA #1 (47%), and sgRNA#2 had the lowest knock-out rate (30%) among the treatments.

## 4. Discussion

As the world scrambles to find alternate sources of protein for food and feed, insects have been identified as a sustainable alternative to provide high-quality protein while minimizing the environmental impact of agriculture and food production [1,45]. However, as with agricultural research in the past, the farming of insects will be more efficient and productive, with increased quality attributes, through accurate and complete genome assemblies. For these reasons, we obtained a reference genome assembly, life staged/sexed transcriptome, and CRISPR-driven genetic engineering tools for *T. molitor*, one of the most intensively farmed insects worldwide.

Our strategy was to extract gDNA from a single male *T. molitor* pupa for the long-read sequencing data, and use long-read, short-read, and long-range data for the best-quality assembly. Expectations that long reads generated from genomic DNA extracted from a single insect would be easier to assemble were soon discarded as the complications of abundant satDNA became apparent. However, we adjusted assembly parameters to get a reasonable draft assembly to begin scaffolding. At the time, we were concerned about the accuracy of long-read data from PacBio RSII sequencing, and thus we incorporated short-read data in a reference-guided assembly to achieve higher base-calling accuracy in a hybrid assembly. Currently this approach is not recommended as the accuracy of long-read data has greatly improved with PacBio HiFi sequencing. Our *T. molitor* assembly is approximately 53% of the predicted genome size of 756.8 ± 9.6 Mb as measured by flow cytometry. It is likely that technologies providing ultra-long reads and/or high accuracy will be necessary to overcome the difficulty of homogeneous satDNA throughout the chromosomes.

Using assembly metrics, we compared our assembly to previous long-read assemblies. Our assembly contained considerably more genes than two previous assemblies and, combined with the BUSCO analysis that found 15% duplication of reference genes, it is likely that our assembly contains duplicated sequence data. We compared the 39,608 genes in *T. molitor* to 16,516 genes identified in *T. castaneum*, and we know that the number of cysteine peptidase genes predicted in this assembly (75) are considerably higher than the 29 that were predicted previously [46]. In addition, synteny analysis with the *T. castaneum* genome found that our largest scaffolds had four sets of fragmented scaffolds. Nonetheless, these fourteen scaffolds contained 92.3% of the total BUSCO reference gene set. A previous assembly had smaller scaffolds (except for one that corresponded to Tcas LGX), and BUSCO genes found in Tcas5.2 LG6 and LG10 were not identified in the largest scaffolds [23]. Although our long-read sequencing was from PacBioRSII, either the amount of coverage or assembly method may have contributed to a more complete draft assembly, as our experience in insect genome sequencing has taught us that having a good long-read draft assembly is crucial to downstream scaffolding.

A short-read analysis found a *T. molitor* satellitome containing 11 satDNA with remarkable sequence conservation (83.8 to 98.6%) occupying 28% of the genome. These satDNA were mapped to the genome assembly scaffolds and were also found in other insect genomes. Repetitive genomic regions, especially those that are highly similar and spread throughout the genome, cause significant technical problems in DNA sequencing and assembly and are mostly omitted or underrepresented in genome assemblies. For that reason, the existing genome assemblies frequently need to be reassessed and updated. As comprehensive and accurate characterization, classification, and annotation of repetitive sequences is an important contribution to the understanding of genomic architecture, many bioinformatics tools, databases, and pipelines have been generated to meet these demands (reviewed in [47]). In particular, strategies combining unassembled (low-coverage) short-read DNA sequences and specialized bioinformatic tools present a completely new concept of large-scale detection and high-throughput analyses, enabling identification of a complete inventory of satDNAs in the genome (i.e., the satellitome), without the need for a genome assembly [41,48,49]. Such approaches are particularly useful as they can provide valuable information regarding the unassembled data and the gaps in the existing assemblies.

An impressive aspect of the genome of *T. molitor* is the contribution of one satDNA in particular. This satDNA has a monomer repeat length of 142 bp, located by FISH in pericentromeric heterochromatin of all chromosomes, and has been estimated to constitute about 50% of metaphase chromosomal length [44,50,51] (Petitpierre et al. 1988, Juan et al. 1990, Petitpierre et al. 1995). Some estimates of the genome occupancy by this satDNA are as much as 60% [52]. The primary location of this satDNA in the pericentromeric heterochromatin of all chromosomes was confirmed by in situ restriction digestion of metaphase chromosomes by endonucleases with recognition sites in the satDNA monomer [53], and it was shown that sequence variants of this satDNA are distributed among all chromosomes [54]. The discrepancy between the older experimental assessments of the 142 bp satDNA content to about 50% of the genome, and the novel assembly-free methods employed in this work averaging its amount to 26.5%, could be due both to the imprecision in the older assessments, and the saturation of the novel pipelines with highly similar repeat units present in high copy-numbers. Subsequent in silico analysis localized 142 bp TmSat1 on a number of scaffolds; however, a significant amount is located throughout the TmoDt5_rgH0X_scaff_79 scaffold, which is replete with this major satDNA of *T. molitor*. While this scaffold is not included in the 14 larger chromosome-scale scaffolds, it is still a significant contribution at 2,386,707 bp and likely could not integrate into the assembly due to the concentration of the major satellite. There is a possibility that these arrays actually originated from different chromosomes and parts of the genome but have been assembled dominantly to this scaffold. However, a significant improvement with respect to the amount of 142 bp TmSat1 presented in this genome assembly has been accomplished. For comparison, in previous scaffold-level assemblies of the *T. molitor* genome, RepeatMasker detected 406 instances of this satDNA across 25 scaffolds (0.08% of the assembly) [23], and 334 instances across 155 scaffolds (0.02% of the assembly) [22]. In Kaur et al. [24], all satDNAs together constituted 0.15% of the assembly, without special distinction of the TmSat1 contribution.

While previous attempts to detect other satDNA in this organism using classical restriction-based methods have failed, employment of the short-read data and TAREAN tool enabled the detection of 10 additional low-copy satDNAs in this organism. While TmSat1 and TmSat7 have been found in other insect species with high sequence identity, other satDNAs only have partial sequence similarity, mostly limited to a certain segment of the monomer sequence. It is known that satDNA monomers sometimes contain conserved segments that exhibit reduced variability with respect to the rest of the monomer sequence. This reduced variability is proposed to be a result of constraints imposed on this sequence segment due to some functional roles, such as DNA–protein interactions. One example is the CENP-B protein, which facilitates centromere formation upon recognizing and binding the conserved CENP-B box, residing within the α-satellite sequence [55]. In this respect, it is possible that diverged variants of *T. molitor* satDNAs exist in other species, with similarity preserved only in a certain segment of the satDNA monomer, potentially involved in some functional interaction.

A transcriptome project with 12 life stages/sexes with 279 million reads was used for gene prediction and genetic engineering. The complete analysis of the transcriptome is outside of the scope of this paper and will be the subject of a future publication, but we performed several analyses to examine differences in gene expression between the different life stages. Genes that were the most statistically different in expression among life stages and sexes had associated GO terms glycosyl and other hydrolases and cytochrome P450s and were likely associated with the diverse metabolism and environmental stress among life stages or between sexes. A highly expressed gene in many of the stages, g38548.t1, encoded a peptide that is conserved in many organisms including bacteria and fungi, where it may have originated. While this is a hypothetical peptide in many organisms, homologs in some have been annotated as a CHK1 checkpoint protein that is a serine/threonine kinase involved in mitosis and DNA damage. Finding a common GO term for viral or transposable element proteins in all samples suggests that these transcripts of foreign genes may have evolved essential functions in this insect. A particular form of TE exaptation, also known as domestication, occurs when TE-encoded proteins or domains become co-opted into functional host proteins, and numerous DNA-binding proteins and transcription factors are known to be derived from transposases (reviewed by [56]).

A novel CRISPR Cas9 system was developed using a plasmid delivery system containing guide RNAs targeting the eye color gene *vermilion* from *T. molitor*, flanking a *T. molitor muscle actin* promoter and EGFP marker [3]. We determined that using a knock-in DNA construct as a circular plasmid containing both guide RNAs and EGFP provided a marker for successful CRISPR. This rationale was used to design the CRISPR experiment for *T. molitor* in this study, and successfully created both a knock-out eye color phenotype and knock-in EGFP marker expression. For the knock-out eye color screening, it was easy to identify the phenotype in a newly hatched larva, but was more difficult in later stages as they tan during the second instar. Although there was evidence of knock-out of the *Tm vermilion* gene, there still was gradual pigmentation that resulted in an eye color similar to wild type. A similar phenomenon was observed in adulthood, as it was much harder to identify the knock-out phenotype in older adults. For the knock-out efficiency, we were only able to identify the complete knock-out eye color phenotype in G_0_ larvae. Mosaic phenotypes (partial knock-out) were observed only when found in stronger somatic knock-out events. As a result, we only recorded complete knock-out events in G_0_s but not the knock-out rate.

The knock-in phenotype of EGFP expression was clear and easy to screen. Originally, we labeled the EGFP-positive G_0_s as “somatic knock-in”. However, many G_0_ larvae without sgRNA in the injection mixture also had EGFP expression. Between the sgRNA and non-sgRNA injections, EGFP-positive G_0_s had the same expression pattern as in muscle tissue and sometimes expressed in a large area of the body as well. Ultimately, we referred to all of them as transient expression G_0_s for a more accurate description. The transient expression may be due to the eggs injected in the early embryonic development stage. There were no knock-out and no EGFP-positive offspring from non-sgRNA treatments in G_2_ individuals, which is further evidence that the EGFP observed in insects injected without guides was transient expression. However, because of this, we cannot identify the differences between transient expression and somatic knock-in in G_0_s.

Knock-out self-crosses did not always provide knock-out G_1_s. In fact, two out of three single sgRNA treatments lacked knock-out G_1_s. However, the knock-out rate should be higher than reported because this knock-out of eye color was a recessive phenotype, and offspring from wild type eye color were not maintained but potentially may have had allele knock-out events. Furthermore, knock-out G_2_s were recovered from EGFP-positive G_0_s from all sgRNA treatments. G_1_ self-crosses with a knock-out rate of 61% were much better than knock-out G_2_ self-crosses (16%), indicating that EGFP-expressing G_0_s could be used to recover both phenotypes and reduced screening time and additional crosses in the future. More interestingly, the data of using one guide vs. three, especially with sgRNA #2 with the lowest knock-out rate (30%) but higher transient expression rate in G_0_ (69%) and knock-in rate in G_1_ (30%), suggests that too much CRISPR cutting is problematic or somehow detracts from successful knock-ins, such as CRISPR excessive cutting of the knock-in construct plasmid itself, hyper cutting of the target genome sequence, or other issues.

Using flow cytometry, we found that the genome sequence of *T. molitor* is longer than previous determinations due to the accumulation of highly similar satDNA sequences. While most assemblies contain a large portion of coding sequences, information on how satellites can affect gene function, as well as more complete scaffolding to reveal regulatory regions, will be necessary to fully understand the biology and evolution of this insect. Sequencing such an important insect to the food and feed industries will promote the development of mealworms and other farmed insects for supplemental feed in agriculture.

## 5. Conclusions

Identification of alternate sources of protein for food and feed is urgently needed. Insects meet this need, being a high-quality protein with production that has minimal effects on the environment. To develop specific insect-based food and feed products for applications, genome sequencing and genetic engineering tools are needed for farmed insects. Acquiring the complete reference genome assembly of a major farmed insect, *T. molitor*, is complicated by largely homogeneous satellite DNA sequences present in high copy numbers. However, the *T. molitor* assembly includes most of the coding regions and will support projects such as population genomics and genetic enhancements. Demonstration of a novel CRISPR Cas9 plasmid to knock-out the eye color gene *Tm vermilion* and knock-in an EGFP marker gene will enable the rapid engineering of mealworm and other insects for downstream applications.

## Figures and Tables

**Table 1:** **Metrics for the assembly of the *T. molitor* genome.** The draft assembly from CANU was used to partially scaffold with Chicago/HiRise. The output from the Chicago/HiRise was used as a reference for a guided assembly of MiSeq reads in SeqManNGen. That hybrid assembly was scaffolded with Hi-C in HiRise for a final assembly.

Assembly	#Scaffolds	Total Bases	N_50_	Longest Scaffold	BUSCO (%)
CANU	7484	417,676,750	103,701	1,144,088	96.1
Chicago/HiRise	2364	423,052,750	2,013,304	9,264,513	96.2
Hybrid	1293	400,747,566	2,120,994	9,265,900	96.3
Hi-C/HiRise	1031	400,765,366	26,202,000	44,869,262	98.3

**Table 2:** **Comparison of *T. molitor* genome assemblies using long-read technology.** Assembly metrics include ours (this study, Tmo), GCA_907166875.3 [23], and GCA_027725215.1 [24].

Metrics	Tmo (This Study)	GCA_907166875.3 [23]	GCA_027725215.1 [24]
Total length (Mb)	401	288	258
Percent of genome	53	38	33
Tissue	single male pupa	single male pupa	head and legs
Sequencing technology	PacBio RSII P6	Illumina PCR-free, PromethION	Oxford Nanopore MinION; Illumina NovaSeq
Predicted #genes	35,281	21,418	19,830
Number of scaffolds	1031	111	1986
Scaffold N_50_ (Mb)	26.2	21.9	20.8
Scaffold L_50_	7	6	6
Scaffolding technology	Dovetail Chicago/Hi-C	Dovetail Hi-C	n/a

**Table 3:** ***T. molitor* satellitome.** Primary characteristics of the 11 satDNAs constituting the satellitome of *T. molitor*.

satDNA	Assembly-Free TAREAN Analysis		Genome Assembly				
	Monomer Length (bp)	Abundance (%)	Number of Monomers	Number of Scaffolds Occupied	Average Monomer Identity (%)	bp Occupied	% of the Assembly
TmSat1	142	26.50	11654	36	98.1	1,654,228	0.413
TmSat2	180	0.45	784	6	96.2	141,131	0.035
TmSat3	325	0.38	2360	19	98.6	765,823	0.191
TmSat4	245	0.26	637	13	87.2	155,006	0.038
TmSat5	364	0.15	781	33	90.4	282,105	0.070
TmSat6	227	0.11	157	10	92.1	35,682	0.009
TmSat7	189	0.08	89	6	97.0	16,800	0.004
TmSat8	735	0.05	127	4	93.9	92,660	0.023
TmSat9	108	0.02	192	1	83.8	20,750	0.005
TmSat10	150	0.01	28	1	92.6	4,210	0.001
TmSat11	93	0.01	6	1	86.8	537	0.0001

## Data Availability

All data was deposited at NCBI, including the *T. molitor* genome assembly—JAWJDX010000000, genomic DNA sequences submitted to SRA—SUB13823713, and the transcripts from life stages were submitted to SRA—PRJNA1012330.

## Supplementary Materials

The following supporting information can be downloaded at: https://www.mdpi.com/article/10.3390/genes14122209/s1, Table S1. Metrics of transcripts from life stages/sexes of *T. molitor*; Table S2. Three sgRNAs selected from the *Tm vermilion* gene for use in CRISPR/Cas9 injections; Table S3. Results of NCBI GenBank database searches with 11 satDNAs of *T. molitor*; Table S4. Statistics on the number of genes expressed > 1000 RPKM in the different *T. molitor* life stages or sexes; Table S5. *T. molitor embryo* microinjections, hatch rate, and transient expression rate; Table S6. Results of the *T. molitor* self-crossed knock-out phenotype screen; Table S7. Results of the *T. molitor* G_1_ EGFP knock-in phenotype screen. Figure S1. Synteny graphs of BUSCO mapped reads of *T. castaneum* (Tcas5.2) to *T. molitor* assemblies Tmo_v3 (GCA_907166875.3) and Tmo (this paper); Figure S2. ShinyGO representation of the GO terms enriched in the differentially expressed genes among all life stages (*p* < 0.05); File S1. Information on the gene annotation of the *T. molitor* assembly; File S2. Fasta file of *T. molitor* satDNA sequences.
