# Supplementary material for: The Genome of the Yellow Mealworm, Tenebrio molitor: It’s Bigger Than You Think
Source: Genes (Basel). 2023 Dec 14;14(12):2209. doi: 10.3390/genes14122209 (PMC10742464; doi:10.3390/genes14122209)
Supplement: Supplementary file 1 [file genes-14-02209-s001.zip › Table S1.pdf]

Table S1. Metrics of transcripts from lifestages/sexes of *T. molitor*.

Lifestage	Abbreviation	Biological replicates	Total Reads/lifestage
Eggs	eggs	4	21,988,095
early larvae(<1mo)	el	4	19,963,350
middle larvae (1 to 2 mo)	ml	4	26,316,029
large larvae (2 mo to prepupae)	ll	4	26,089,271
early female pupae	efp	4	29,764,923
early male pupae	emp	4	26,854,148
late female pupae	lfp	4	25,910,537
late male pupae	lmp	2	6,690,083
early female adult	efa	4	23,910,370
early male adult	ema	4	23,748,255
late female adult	lfa	4	26,658,438
late male adult	lma	3	21,158,006
TOTAL		45	279,051,505
