# Supplementary material for: The Genome of the Yellow Mealworm, Tenebrio molitor: It’s Bigger Than You Think
Source: Genes (Basel). 2023 Dec 14;14(12):2209. doi: 10.3390/genes14122209 (PMC10742464; doi:10.3390/genes14122209)
Supplement: Supplementary file 1 [file genes-14-02209-s001.zip › Table S2. CRISPR .pdf]

Table S2. Three sgRNAs selected from the *Tm vermilion* gene for use in CRISPR/Cas9 injections.

sgRNA	Sequence + PAM
# 1	GAACAACTGGGTGAAGAAGATGG
# 2	GGGCAAGATAGTGAACAACTGGG
# 3	ACTTACGAGGTTCTTCTAACAGG
