# Supplementary material for: The Genome of the Yellow Mealworm, Tenebrio molitor: It’s Bigger Than You Think
Source: Genes (Basel). 2023 Dec 14;14(12):2209. doi: 10.3390/genes14122209 (PMC10742464; doi:10.3390/genes14122209)
Supplement: Supplementary file 1 [file genes-14-02209-s001.zip › Table S3.pdf]

Table S3. Results of NCBI GenBank database searches with 11 satDNAs of *T. molitor*.

	WGS insecta (4235 databases)				NCBI insecta nucleotide collection			
**satDNA**	**Species**	**Order**	**Query cover (%)**	**Identity (%)**	**Species**	**Order**	**Query cover (%)**	**Identity (%)**
**TmSat1 (142 bp)**	*Solenopsis invicta*	Hymenoptera	100	100	*Tenebrio obscurus*	Coleoptera	99	78.3
	Periplaneta americana	Blattodea	100	98.6				
	Zophobas morio	Coleoptera	100	100				
**TmSat2 (180 bp)**					*Camptogramma bilineata*	Lepidoptera	21	89.7
**TmSat3 (325 bp)**	*Phosphuga atrata*	Coleoptera	28	77.6	*Phosphuga atrata*	Coleoptera	29	78.7
	Drosophila melanica	Diptera	11	97.3	*Aelia acuminata*	Hemiptera	12	90.0
	Drosophila micromelanica	Diptera	10	100	*Bellardia bayeri*	Diptera	18	85.4
**TmSat4 (245 bp)**	*Podabrus alpinus*	Coleoptera	36	76.6	*Podabrus alpinus*	Coleoptera	64	75.5
	Hydrotaea cyrtoneurina	Diptera	24	91	*Cantharis rustica*	Coleoptera	25	90.0
					Cantharis nigra	Coleoptera	17	88.1
					Cantharis rufa	Coleoptera	17	88.1
					Bemisia tabaci	Hemiptera	15	89.5
					Rhagonycha fulva	Coleoptera	17	88.1
					Hydrotaea cyrtoneurina	Diptera	25	88.0
					Salpingus planirostris	Coleoptera	11	100
					Agonum fuliginosum	Coleoptera	24	80.3
**TmSat5 (364 bp)**	*Neruda aoede*	Lepidoptera	17	83.1	*Miltochrista miniata*	Lepidoptera	11	88.4
					Yponomeuta plumbellus	Lepidoptera	10	89.5
**TmSat6 (227 bp)**	*Periplaneta americana*	Blattodea	95	93.5	*Melinaea menophilus*	Lepidoptera	17	92.7
					Synanthedon formicaeformis	Lepidoptera	22	82.7
					Chloroclysta siterata	Lepidoptera	19	84.4
					Melinaea marsaeus rileyi	Lepidoptera	16	89.7
**TmSat7 (189 bp)**	*Dinoponera quadriceps*	Hymenoptera	100	100				
	Periplaneta americana	Blattodea	88	97.1				

**TmSat8 (735 bp)**	*Zophobas atratus*	Coleoptera	18	82.3	*Dilophus febrilis*	Diptera	14	74.1
	Tribolium madens	Coleoptera	16	75.6	*Cantharis rustica*	Coleoptera	13	73.7
	Tribolium freemani	Coleoptera	13	73.9	*Acrobasis suavella*	Lepidoptera	12	74.2
	Latheticus oryzae	Coleoptera						
**TmSat9 (108 bp)**								
**TmSat10 (150 bp)**	*Zophobas atratus*	Coleoptera	56	85.7	*Tribolium freemani*	Coleoptera	54	76.8
	Tribolium confusum	Coleoptera	78	73.7	*Vespula vulgaris*	Hymenoptera	30	86.7
	Tribolium freemani	Coleoptera	54	76.8	*Vespula germanica*	Hymenoptera	30	86.7
	Tribolium castaneum	Coleoptera	54	76.8	*Perizoma affinitatum*	Lepidoptera	25	89.4
	Latheticus oryzae	Coleoptera	52	79.5	*Thymelicus sylvestris*	Lepidoptera	30	87.0
	Cynaeus angustus	Coleoptera	48	78.0	*Myopa testacea*	Diptera	35	83.0
					Ligdia adustata	Lepidoptera	25	89.5
**TmSat11 (93 bp)**								
