# Supplementary material for: The Genome of the Yellow Mealworm, Tenebrio molitor: It’s Bigger Than You Think
Source: Genes (Basel). 2023 Dec 14;14(12):2209. doi: 10.3390/genes14122209 (PMC10742464; doi:10.3390/genes14122209)
Supplement: Supplementary file 1 [file genes-14-02209-s001.zip › Table S4.pdf]

Table S4. Statistics on the number of genes expressed >1,000 RPKM in the different *T. molitor* lifestages or sexes.

Developmental stage	Number of genes (>1000 RPKM)	Gene with highest expression	Description	RPKM
eggs	100	g38548.t1*	hypothetical protein TcasGA2_TC034988, partial	132,696.576
el	99	g22275.t1	no annotation	245,327.223
ml	115	g22275.t1	no annotation	138,457.527
ll	104	g38548.t1	hypothetical protein TcasGA2_TC034988, partial	118,368.951
emp	89	g39335.t1	no annotation	133,624.082
lmp	105	g20218.t1	no annotation	114,715.827
ema	122	g38548.t1	hypothetical protein TcasGA2_TC034988, partial	152,189.755
lma	137	g22275.t1	no annotation	155,107.997
efp	146	g38548.t1	hypothetical protein TcasGA2_TC034988, partial	165,600.659
lfp	145	g22275.t1	no annotation	164,052.483
efa	97	g38548.t1	hypothetical protein TcasGA2_TC034988, partial	93,422.419
lfa	124	g22275.t1	no annotation	209,018.561

- Amino acid sequence: MVGSGLPCCNGLTGNQGSIPEREPEKRLPHSRKAAGAQITHSRHGEVVTKNNDTGLIRGPVIGMSTL
