# Supplementary material for: The Genome of the Yellow Mealworm, Tenebrio molitor: It’s Bigger Than You Think
Source: Genes (Basel). 2023 Dec 14;14(12):2209. doi: 10.3390/genes14122209 (PMC10742464; doi:10.3390/genes14122209)
Supplement: Supplementary file 1 [file genes-14-02209-s001.zip › Table S5. T molitor embryo microinjection.pdf]

Table S5. ***T molitor embryo*** **microinjections, hatch rate, and transient expression rate.** Injections with sgRNA # 1, 2, or 3, and 1/2/3 are detailed in methods; two groups were injected without sgRNAs as controls.

sgRNA	sgRNA (pmol/mL)	#eggs	Hatched	Hatch rate (%)	G_0 transient expression rate (%)
# 1/2/3	18	480	182	38	13
# 1	6	508	290	57	58
# 2	6	509	264	52	69
# 3	6	537	251	47	50
none	N/A	185	91	49	27
none	N/A	540	194	36	51
