# Supplementary material for: The Genome of the Yellow Mealworm, Tenebrio molitor: It’s Bigger Than You Think
Source: Genes (Basel). 2023 Dec 14;14(12):2209. doi: 10.3390/genes14122209 (PMC10742464; doi:10.3390/genes14122209)
Supplement: Supplementary file 1 [file genes-14-02209-s001.zip › Table S6. Results of the T molitor self-crossed .pdf]

Table S6. Results of the *T molitor* self-crossed knock-out phenotype screen.

sgRNA	# Knock-out self-crosses	# of white eye in G_1	# of G_1 self-crosses	# of white eye in G_2	Knock-out Rate (%)
# 1, 2, 3	4	2	9	5	54
# 1	7	2	12	7	47
# 2	11	0	9	6	30
# 3	3	0	6	4	44
none	1	0	1	0	0
