# Supplementary material for: The Genome of the Yellow Mealworm, Tenebrio molitor: It’s Bigger Than You Think
Source: Genes (Basel). 2023 Dec 14;14(12):2209. doi: 10.3390/genes14122209 (PMC10742464; doi:10.3390/genes14122209)
Supplement: Supplementary file 1 [file genes-14-02209-s001.zip › Table S7. Results of the T molitor G1 EGFP .pdf]

Table S7. Results of the *T molitor* G_1 EGFP knock-in phenotype screen. In the injections with either sgRNA # 1, 2, 3, or 1/2/3, or no sgRNA, the parameters are provided for the number of individuals in the G_1 knock-in out-crosses and number of crosses with EGFP for G_2, and the number of individuals in the knock-out self-crosses, and # of crosses with EGFP for G_2.

sgRNA	# of knock-in out-crosses	# of crosses w/ E+ G_1	# of knock-out self-crosses	# of crosses w/ E+ G_1	Knock-in rate (%)
3 sgRNA	9	0	4	0	0
sgRNA#1	12	2	7	0	10
sgRNA#2	9	6	11	0	30
sgRNA#3	6	2	3	0	22
none	1	0	0	0	0
