# Supplementary figures and images for: The Genome of the Yellow Mealworm, Tenebrio molitor: It’s Bigger Than You Think
Source: Genes (Basel). 2023 Dec 14;14(12):2209. doi: 10.3390/genes14122209 (PMC10742464; doi:10.3390/genes14122209)

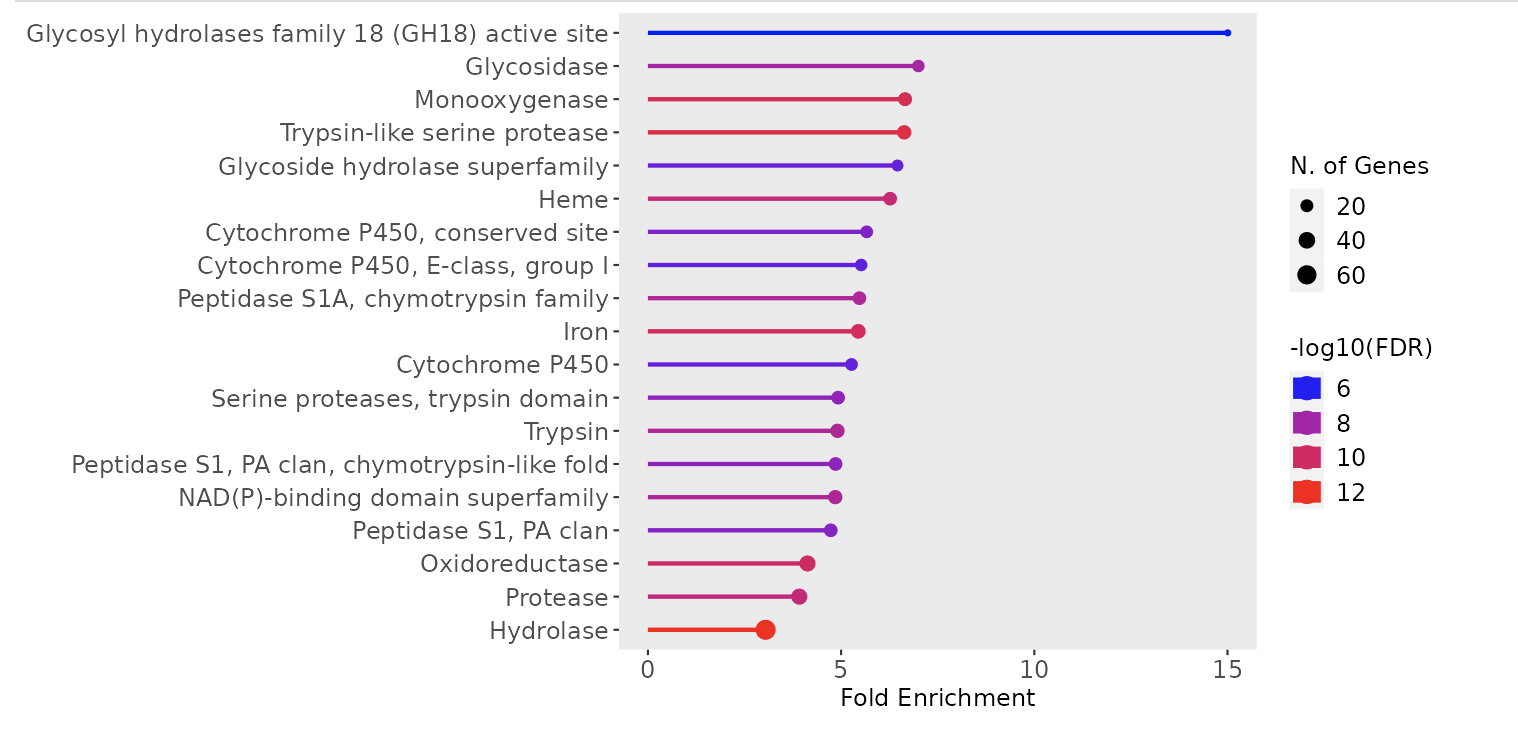

Supplement: Supplementary file 1 [file genes-14-02209-s001.zip › Figure S2.png]

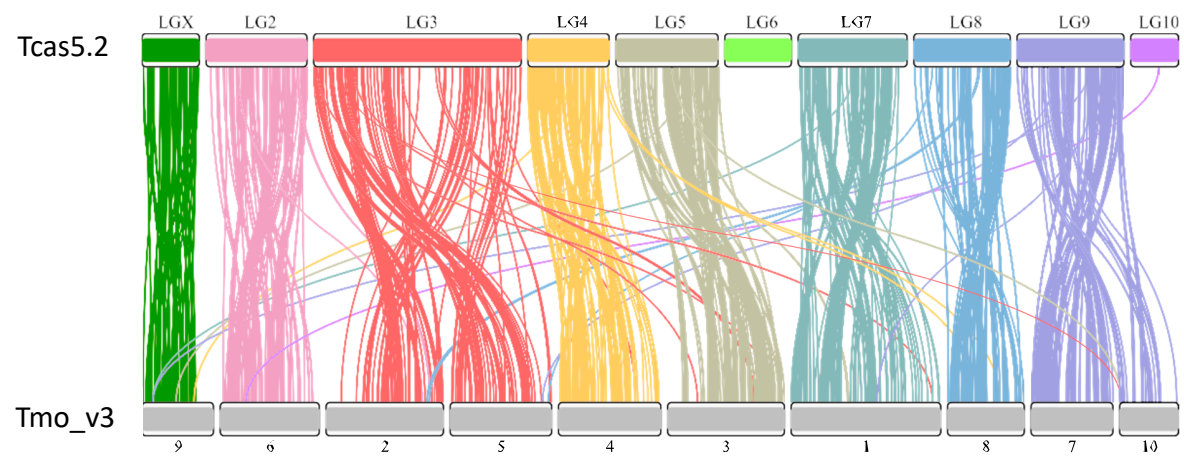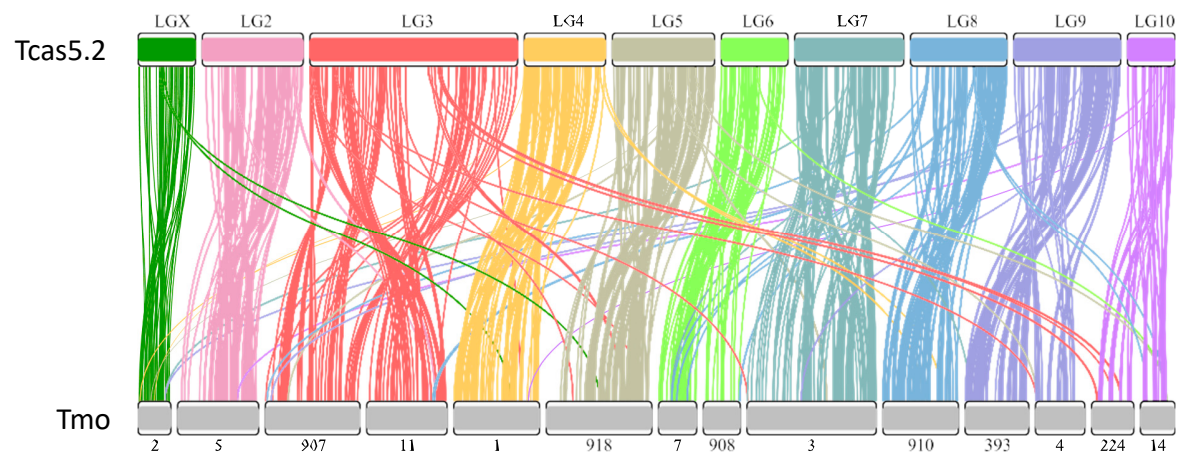

Supplement: Supplementary file 1 [file genes-14-02209-s001.zip › Figure S1.pdf]
